# Agricultural Task Not Predictive of Children’s Exposure to OP Pesticides

**DOI:** 10.1289/ehp.112-a865c

**Published:** 2004-11

**Authors:** Richard A. Fenske, John C. Kissel, Jeffry H. Shirai, Cynthia L. Curl, Kit Galvin

**Affiliations:** Department of Environmental and Occupational Health Sciences, School of Public Health and Community Medicine, University of Washington, Seattle, Washington, E-mail: rfenske@u.washington.edu

Coronado et al. (2004) reported that the agricultural task of plant thinning by adults was associated with higher urinary pesticide metabolite concentrations in children. Their analysis was based on data from a 1999 study of farmworkers in the Yakima Valley of Washington State (Curl et al. 2002; Thompson et al. 2003). Their conclusion was based on a finding that one of the three dimethyl dialkylphosphate (DAP) metabolites of the organophosphorus (OP) pesticides—dimethylthiophosphate (DMTP)—was more frequently detected among children living in the same household with adult farmworkers who reported having thinned plants compared with children living in the same household with farmworkers who did not report thinning (92% vs. 81%, respectively).

We examined the same data set to determine if the actual urinary pesticide metabolite concentrations, rather than simply the frequency of metabolite detection, differed between these groups of children. We used log-transformed data and the independent *t*-test (equal variance assumption) to determine differences between geometric mean metabolite concentrations. We found no significant differences between children of thinners versus non-thinners for any of the three DAP metabolites. Geometric mean values for DMTP were 6.13 μg/L for 136 children of thinners and 5.27 μg/L for 75 children of non-thinners (*p* = 0.41). Furthermore, we did not find a significant difference between these groups for the sum of the dimethyl DAP metabolites (geometric means of 0.097 vs. 0.083 μmol/L; *p* = 0.33).

Coronado et al. (2004) also suggested that children of thinners may receive higher exposures than children of pesticide handlers (mixers, loaders, applicators). We compared the children of thinners to children of handlers, excluding the 28 children for whom the corresponding adult farmworker reported both thinning and handling. No differences were observed between these groups for any of the three DAP metabolites. Geometric mean values for DMTP were 6.47 and 6.05 μg/L, respectively (*p* = 0.81), and 0.10 and 0.096 μmol/L, respectively, for the sum of the dimethyl DAP metabolites (*p* = 0.78). It is not surprising that the child population in this study exhibited high frequencies of detection of the DAP metabolites. The most recent study by the Centers for Disease Control and Prevention (Barr et al. 2004) found that 87% of U.S. children 6–11 years of age had one or more of the dimethyl DAP metabolites detected in their urine.

We conclude from our analysis of this data set that *a*) children living in households with thinners did not exhibit higher OP pesticide exposures than children living in households with workers in other agricultural task categories; and *b*) OP pesticide exposures did not differ between children of thinners and children of pesticide handlers. We further conclude that frequency of detection is an inadequate exposure metric for urinary pesticide metabolites that are detected with high frequency, and that its use independent of metabolite concentration data can prove misleading. We recommend that future analyses of children’s pesticide exposure focus on measured metabolite concentrations rather than the simple presence or absence of metabolites in biological samples.
